# Supplementary material for: Electrically controlled nonvolatile switching of single-atom magnetism in a Dy@C84 single-molecule transistor
Source: Nat Commun. 2024 Mar 19;15:2450. doi: 10.1038/s41467-024-46854-z (PMC10951203; doi:10.1038/s41467-024-46854-z)
Supplement: Supplementary file 1 — Supplementary Information [file 41467_2024_46854_MOESM1_ESM.pdf]

Supplementary Information for

Electrically controlled nonvolatile switching of single atom magnetism in a Dy@C_{84} single-molecule transistor

Feng Wang[1,2,#], Wangqiang Shen[3,4,#], Yuan Shui[5,#], Jun Chen[1,2], Huaiqiang Wang[6], Rui Wang[1], Yuyuan Qin[1], Xuefeng Wang[7], Jianguo Wan[1], Minhao Zhang[1,2*], Xing Lu[3,*], Tao Yang[5,*], Fengqi Song[1,2*].

[1]National Laboratory of Solid State Microstructures, Collaborative Innovation Center of Advanced Microstructures, and School of Physics, Nanjing University, Nanjing 210093, China

[2]Institute of Atom Manufacturing, Nanjing University, Suzhou 215163, China

[3]State Key Laboratory of Materials Processing and Die & Mould Technology, School of Materials Science and Engineering, Huazhong University of Science and Technology, Wuhan 430074, China

[4]School of Materials Science and Engineering, Hefei University of Technology, Hefei 230009, China

[5]MOE Key Laboratory for Non-Equilibrium Synthesis and Modulation of Condensed Matter, School of Physics, Xi'an Jiaotong University, Xi'an, 710049, China

[6]Center for Quantum Transport and Thermal Energy Science, School of Physics and Technology, Nanjing Normal University, Nanjing 210023, China

[7]School of Electronic Science and Engineering and Collaborative Innovation Center of Advanced Microstructures, Nanjing University, Nanjing 210023, China

[#]These authors contributed equally: Feng Wang, Wangqiang Shen, Yuan Shui

[*]Corresponding authors. Email: M.Z. (zhangminhao@nju.edu.cn), X. L. (lux@hust.edu.cn), T.Y. (taoyang1@xjtu.edu.cn) and F.S. (songfengqi@nju.edu.cn)

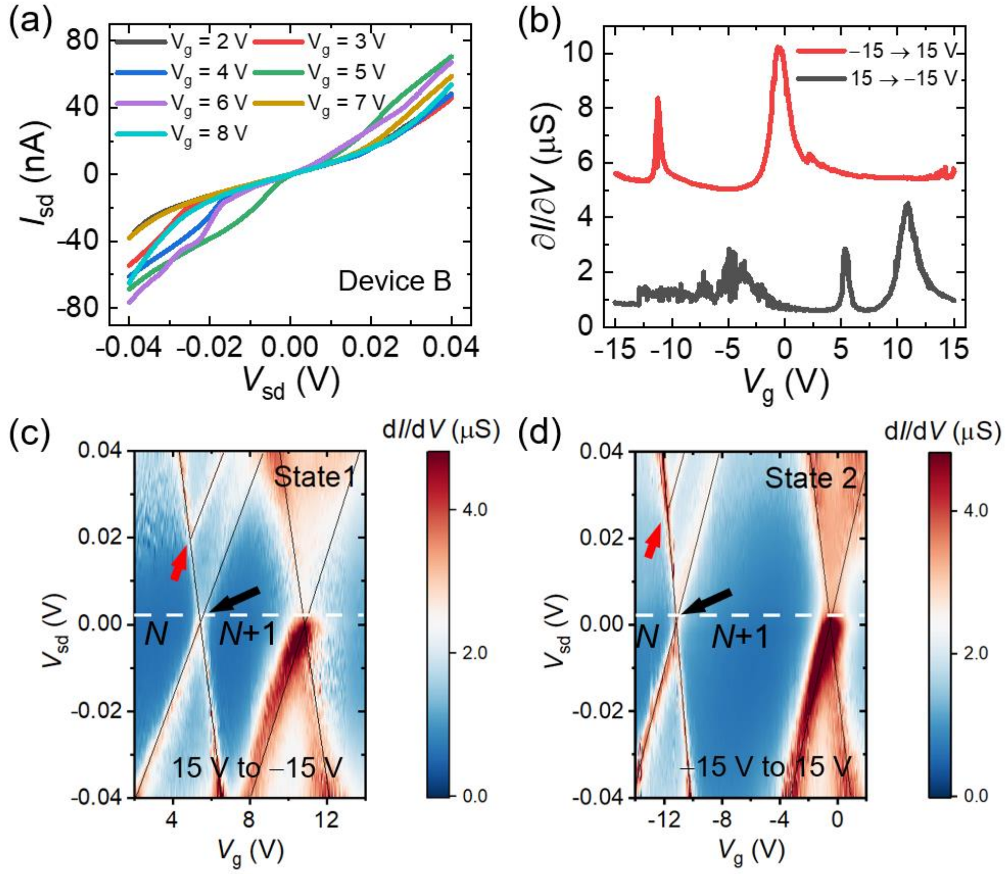

Supplementary Figure 1 Basic electrical transport measurements of device B. (a) The $I_{sd}(V_{sd})$ characteristic curves at different V_g after electromigration. V_g can modulate the current blockade region. (T = 1.8 K; the whole electrical transport measurements in device B were carried out at this cryogenic temperature). (b) The differential conductance dI_{sd}/dV_{sd} as a function of V_g at zero bias voltage V_{sd} = 0 mV extracted from numerical differentiation of the current maps, which exhibits two set of Coulomb oscillation patterns. The traces have been offset vertically for clarity. (c, d) The two-dimensional dI_{sd}/dV_{sd} maps corresponding to the black line (V_g = 2 – 14 V) and red line (V_g = −14 – 2 V) in (b), respectively, plotted as a function of V_g and V_{sd} in device B. The Coulomb diamond, marked by the solid line, represents a single electron tunneling through the central molecule. The intersections of these Coulomb edges at zero bias are

charge degeneracy points (resonant tunneling points). The gate efficiency factor α is 0.0076 for (c) and 0.008 for (d). The energies of excitons marked by red arrows are approximately 20 meV in (c) and 27 meV in (d), respectively. The excited states can presumably be assigned to the longitudinal vibration of the Dy ion coupled to the carbon cage[1], rather than an intrinsic vibrational mode of the carbon cage.

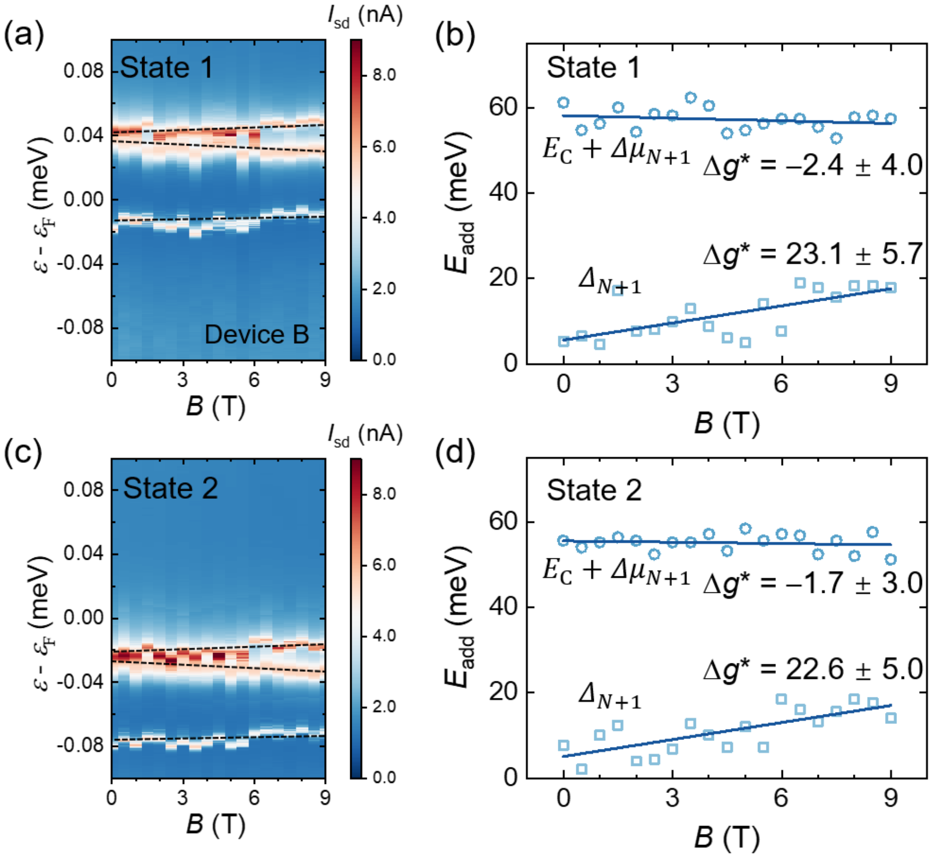

Supplementary Figure 2 Zeeman effect of device B. (a, c) Coloured maps of the current for state 1 (a), and state 2 (c), as a function of an applied magnetic field and relative energy tuned by V_g (converting V_g to energy using the gate efficiency factor α) in device B. The bias voltage is fixed at 2 mV. The maps demonstrate clear evidence of Zeeman effect and a level splitting behavior of the (N+1) charge state. The colour bar represents the current intensity. (b, d) Addition energy and level splitting for the (N+1) charge states as a function of magnetic field of state 1 (b) and state 2 (d). The addition energy defined as $E_{add}(N) = \mu_{N+1} - \mu_N = E_C + E_{N+1} - E_N$ (where μ is the chemical potential, E_C is the charging energy, E_{N+1} and E_N are energy level of the (N+1) and N charge states) is calculated by measuring the spacing between consecutive Coulomb peaks. $\Delta\mu_{N+1}$ is the energy level spacing between the (N+1) and N charge states, and Δ_{N+1} is the energy level splitting of the (N+1) charge state. We fit the data to obtain the

effective g-factor. The g^* value for the level splitting Δ_{N+1} of the (N+1) charge state reaches approximately 20, which is comparable to the splitting in the ground state |±15/2> of Dy^{3+}. The slope of the addition energy E_{add} with respect to B depends on the relative spin orientation between two neighboring charge states. The extracted g^* value from the slope of $\Delta\mu_{N+1}$ is approximately -2, indicating that the ground state of the N charge state with magnetic moment of ~8 μ_B may originates from either the excited state of Dy^{3+} or more likely a hybridized state with carbon cage.

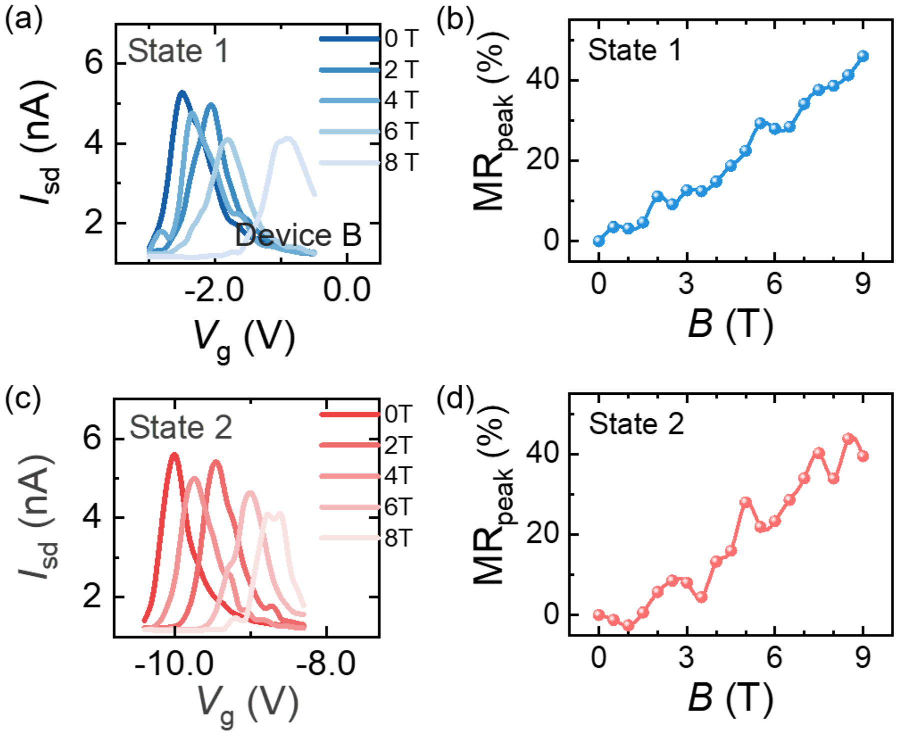

Supplementary Figure 3. Evolution of peak amplitude and MR of the resonance tunneling point for the *N* charge state in device B. (a, c) The resonance tunneling peak of state 1 and state 2 for the *N* charge state ($V_g \sim -2$ V for state 1 and $V_g \sim -10$ V for state 2, indicated by black arrows in Supplementary Figure 1(c, d)) measured under different magnetic fields (B = 0, 2, 4, 6, 8 T) at a fixed bias voltage (V_{sd} = 2 mV). The presence of the magnetic field results in suppression of the peak amplitude. (b, d) Magnetic field dependence of MR extracted from peaks' amplitudes of the resonance tunneling point in (a, c) for state 1 (b) and state 2 (d). The MR of state 1 can reach 45% at B = 9 T, and that of state 2 is 40%. There is small variation in the MR properties of the two molecular states. According to the proposed metal-cage hybrid state, the degree of hybridization will affect the MR properties. The *N* charge state exhibits a small MR value and MR variation, which reveal a low degree of hybridization between the Dy and cage components.

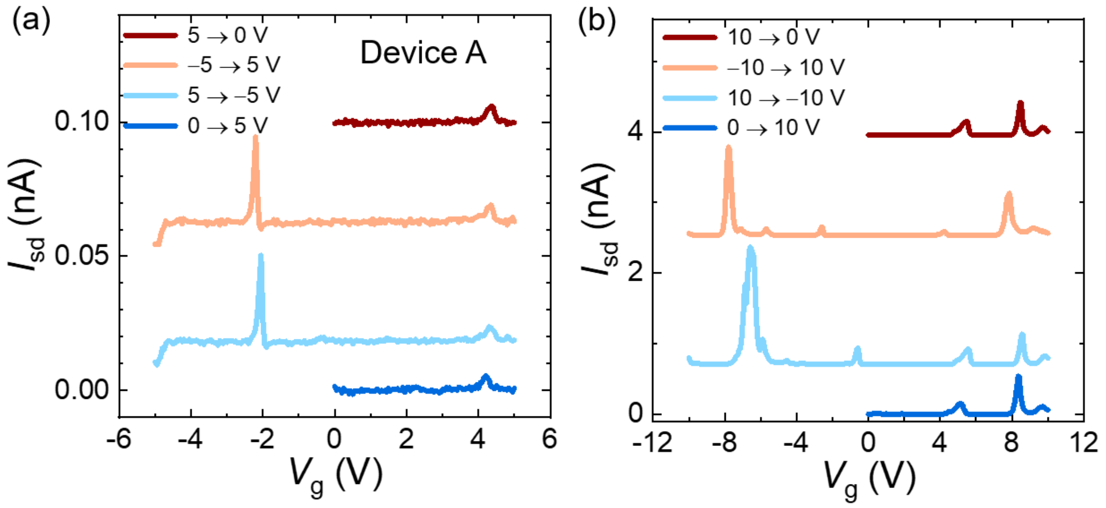

Supplementary Figure 4. Gate-tunable molecular states of device A. (a, b) Original data of I_{sd} as a function of V_g in a range ±5 V (a) and ±10 V (b) swept back and forth for a fixed V_{sd} of 2 mV. The states remain unchanged within a gate voltage range of 5 V, but undergo switching within a range of 10 V.

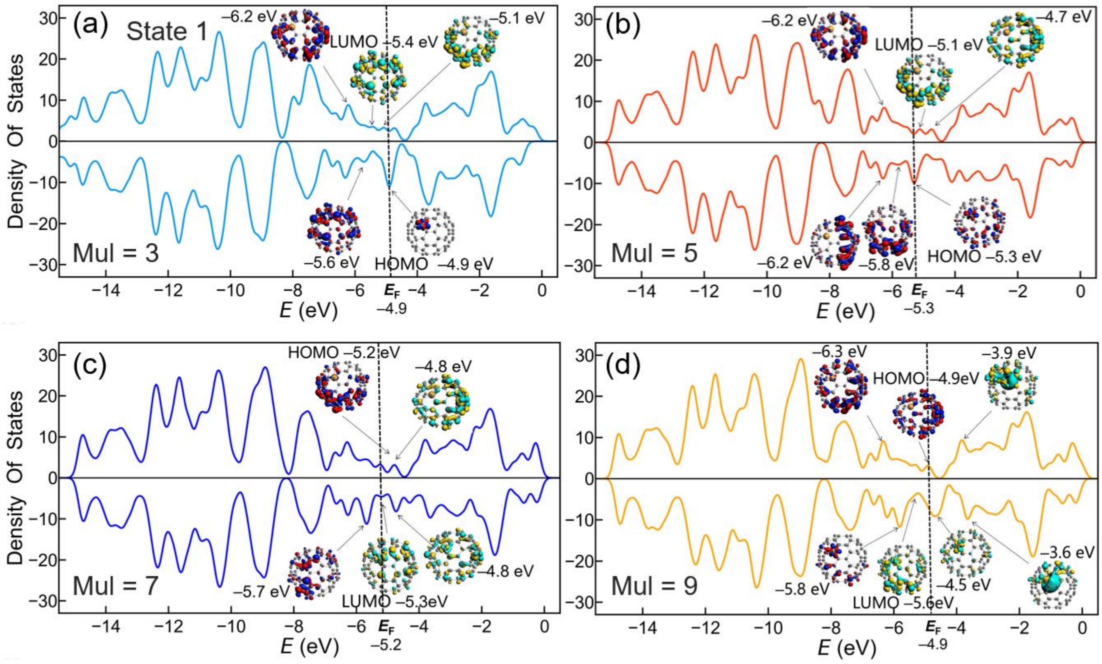

Supplementary Figure 5. The DOS for state 1 in neutral free Dy@C_{84} molecules under different spin multiplicities. (a-d) Under different spin multiplicities Mul = 3 (a), Mul = 5 (b), Mul = 7 (c), and Mul = 9 (d), the calculated DOSs of neutral Dy@C_{84} molecule are plotted. Positive (negative) values denote spin-up (spin-down) electrons. The figure delineates the frontier molecular orbitals (FMOs) of the Dy@C_{84} molecule, highlighting that several molecular orbitals in proximity to the HOMO are engendered through the hybridization of the orbitals between the Dy atom and the C_{84} cage. Notably, in the cases of Mul = 3 and Mul = 5, the predominance of the Dy 4*f* orbitals in contributing to the HOMO is shown.

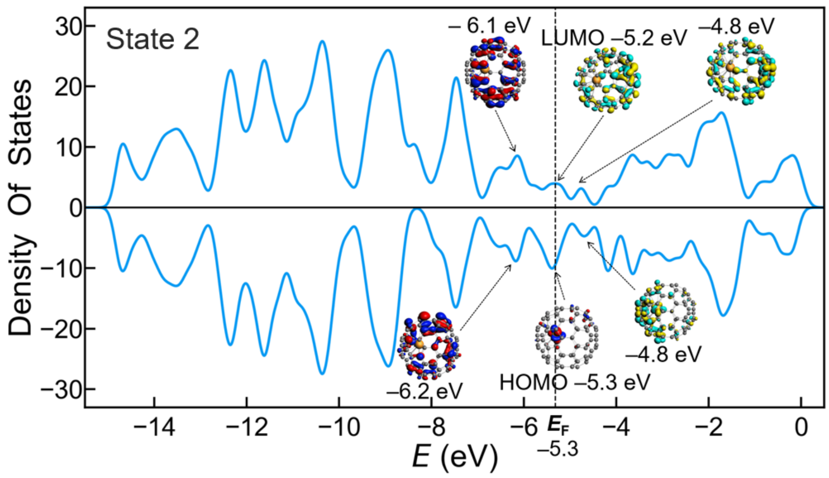

Supplementary Figure 6. DOSs of state 2 in neutral free Dy@C84 molecules. As a comparative analysis, the DOSs pertaining to state 2 of the ground state in the neutral Dy@C84 molecule was meticulously computed (Mul = 5), allowing the presence of metal-cage hybrid orbitals to be discerned within the FMOs.

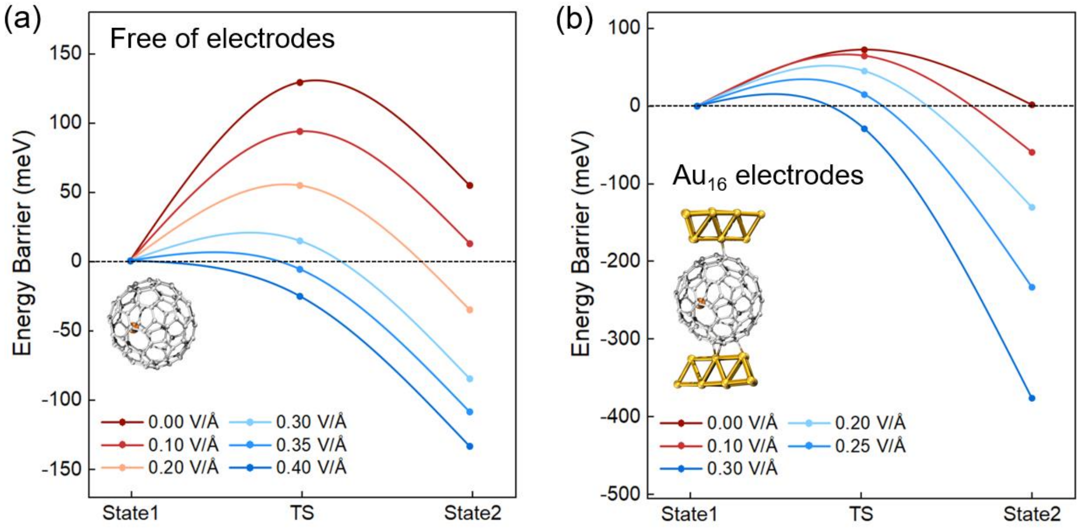

Supplementary Figure 7 Energy barriers under different gate electric fields in Dy@C$_{84}$. (a, b) Calculated energy barrier under different gate electric fields between the bistable states free of electrodes (a) and coupled to Au electrodes (b). The interaction between the molecule and the Au electrodes leads to significant reductions in the energy difference and the barrier. The gate electric field can effectively lower the energy barrier, thereby enhancing the transition probability between the two molecular states.

Supplementary Table 1 Composition in terms of Dy and cage orbitals for the HOMO and LUMO of the bistable states from theoretical calculation.

	State 1	State 2
HOMO	5.1% cage + 94.9% Dy	5.6% cage + 94.4% Dy
LUMO	36.1% cage + 63.9% Dy	37.3% cage + 62.7% Dy

Supplementary Table 2 Energy differences between the two molecular states and energy barriers of bistable switching under different conditions. Charge transfer and the interaction with electrodes play an important role. The calculation results reveal significant disparities in energy and energy barrier between the bistable states for different charge states. Adding an integer number of electrons will significantly increase the energy barrier, while reducing the number of electrons may decrease the barrier. Moreover, the energy difference between two bistable states varies considerably with the charge state.

	Conditions	Energy differences (meV)	Barrier (meV)
Charge transfer	Neutral	+61	+145
	Charge = +1	+120	+134
	Charge = +2	+87	+125
	Charge = –1	–1	+246
	Charge = –2	+103	+266
Interaction with Au electrodes	Au_{16} electrode	+2	+92

Supplementary Note 1 The relationship between effective magnetic moment (μ) and effective *g*-factor (g^*)

Theoretical values of the effective magnetic moment of the free Dy^{3+} ion can be obtained based on the relationship:

$$\mu = \mu_{\mathrm{B}} g_J J \quad (1)$$

$$g^* = g_J J \quad (2)$$

and $g_J = \frac{3}{2} + \frac{S(S+1) - L(L+1)}{2J(J+1)}$ (3)

For the Dy^{3+} ion with $L = 5$, $S = \frac{5}{2}$, $J = \frac{15}{2}$ and $g_J = \frac{4}{3}$, this gives a theoretical effective magnetic moment for the ground state $\left|\frac{15}{2}, \pm\frac{15}{2}\right\rangle$ of $\mu = 10\ \mu_{\mathrm{B}}$. Other values of magnetic moment for the excited states $\left|\frac{15}{2}, \pm m_J\right\rangle$ are $\mu = 10 * \frac{m_J}{15/2} \mu_{\mathrm{B}}$.

Supplementary References

1 Du, Shaoqing, *et al.* Ultrafast rattling motion of a single atom in a fullerene cage sensed by terahertz spectroscopy. *Appl. Phys. Express* **13**, 105002 (2020)
